# Syngas Evolution from CO_2_ Electroreduction by Porous Au Nanostructures

**DOI:** 10.1021/acsaem.0c00301

**Published:** 2020-05-06

**Authors:** Luca Mascaretti, Alessandro Niorettini, Beatrice Roberta Bricchi, Matteo Ghidelli, Alberto Naldoni, Stefano Caramori, Andrea Li Bassi, Serena Berardi

**Affiliations:** † Micro- and Nanostructured Materials Laboratory, Department of Energy, Politecnico di Milano, Via Ponzio 34/3, 20133 Milano, Italy; ‡ Regional Centre of Advanced Technologies and Materials, Faculty of Science, Palacký University, Šlechtitelů 27, 78371 Olomouc, Czech Republic; § Department of Chemical and Pharmaceutical Sciences, University of Ferrara, Via Luigi Borsari 46, 44121 Ferrara, Italy; ∥ Department of Structure and Nano/Micromechanics of Materials, Max-Planck-Institut für Eisenforschung GmbH, Max-Planck Straße 1, 40237 Düsseldorf, Germany

**Keywords:** CO_2_ reduction, pulsed-laser deposition, nanoporous films, Au nanostructures, electrocatalysis

## Abstract

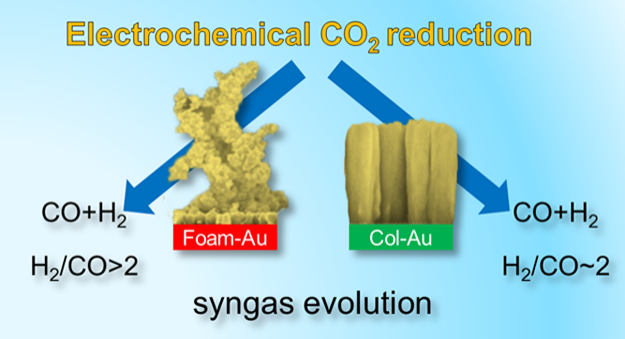

Electrocatalytic reduction of CO_2_ recently emerged as a viable solution in view of changing the common belief and considering carbon dioxide as a valuable reactant instead of a waste product. In this view, we herein propose the one-step synthesis of gold nanostructures of different morphologies grown on fluorine-doped tin oxide electrodes by means of pulsed-laser deposition. The resulting cathodes are able to produce syngas mixtures of different compositions at overpotentials as low as 0.31 V in CO_2_-presaturated aqueous media. Insights into the correlation between the structural features/morphology of the cathodes and their catalytic activity are also provided, confirming recent reports on the remarkable sensitivity toward CO production for gold electrodes exposing undercoordinated sites and facets.

## 1 Introduction

The containment of the greenhouse effect and of serious alterations to ecosystems will likely require not only the net reversal of the currently increasing carbon dioxide (CO_2_) emission trend but also extensive sequestration of this gas from the atmosphere.^1,2^ In this context, the conversion of CO_2_ in alternative fuels by electrochemical reduction represents an intriguing strategy toward the establishment of a virtuous circle,^3−9^ especially if the use of an electrical grid powered by renewable sources is envisaged. Furthermore, provided the use of suitable metallic electrodes, this approach is known to yield different kinds of products,^3,10,11^ some of which (such as carbon monoxide, formate, methane, and methanol) would fit in the currently available infrastructures for the storage and transport of fossil fuels.

At the same time, CO_2_ reduction is a challenging reaction involving several open issues that must be faced in view of a possible industrial implementation. First of all, it is a slow electrochemical process, involving multiple electron and proton transfers, as well as the adsorption of both the gaseous substrate and the reaction intermediates on electrodic metal surfaces.^3^ Furthermore, since CO_2_ electroreduction is most practically achieved in aqueous electrolytes, with reduced environmental impact with respect to organic solvents, the competition of proton reduction to H_2_ is often substantial, jeopardizing the reaction selectivity.^12^ The limitation of the proton reduction pathway is particularly challenging also in view of the slow dissolution rate of CO_2_ in water and its scarce overall solubility (34 mM).^3^ Nevertheless, concomitant hydrogen evolution can be valorized in view of syngas production, i.e., a mixture of CO and H_2_ instrumental in industrial processes, such as Fischer–Tropsch synthesis. In particular, different CO/H_2_ ratios allow for the production of different kinds of chemicals, ranging from liquid fuels (gasoline and diesel) to olefins, methanol, and methane, depending on the catalyst and the reaction conditions.^13−15^

From the mechanistic point of view, the first monoelectronic step of CO_2_ electroreduction is thermodynamically very demanding (−1.9 V vs the normal hydrogen electrode, NHE) since significant geometric rearrangements are involved in the transformation of the linear substrate in the bent radical anion CO_2_^·^^–^. Nevertheless, the coordination of the CO_2_ molecule on electrodic surfaces can effectively mitigate this thermodynamic requirement. Indeed, several metals can effectively stabilize not only CO_2_^·^^–^^16^ but also other key intermediates for the further (multielectronic) reduction reactions. Among them, *CO, *COOH, and *CHO are formed *via* proton–electron transfer mechanisms^17^ (the asterisk denotes a site on the electrodic surface). On the other hand, an optimal binding strength between the intermediates and the metal surface, i.e., not hampering either the coordination or product release, is crucial in terms of the overall catalytic activity, which is usually assessed through volcano plots (Sabatier principle).^17^ As far as CO binding energy is concerned, the top of the volcano is occupied by gold,^4^ which almost selectively forms carbon monoxide as the main CO_2_ reduction product.^3^ Smaller amounts of formic acid^3^ and methanol^18,19^ have been also detected respectively at low and high overpotentials.

Several reports evidenced the importance of nanostructuring the Au-based cathodic interfaces to boost CO formation over the competitive proton reduction in aqueous media. Highly effective nanostructured Au cathodes typically exhibit (i) metastable surface structures,^20^ (ii) engineered high-index facets and features,^21,22^ (iii) local changes in the electric double layer near the cathode surface^23,24^ and/or in the local pH of the electrolyte,^25^ and (iv) undercoordinated sites, including grain boundaries.^25−28^ The latter have been recently identified as the most relevant feature for an efficient CO formation process by Chorkendorff’s group.^29^ Through selective poisoning experiments, the authors could indeed prove that surface sites with high coordination numbers are *ca.* one order of magnitude less active for CO evolution than the undercoordinated sites, confirming the structure sensitivity of the CO_2_ electroreduction process.^29^ Higher selectivity and faster kinetics for CO production by low-coordinated Au(110) electrodes have been also confirmed by online electrochemical mass spectrometry.^30^

Engineered Au morphologies aimed at maximizing CO selectivity have been prepared through most various synthetic strategies, including (i) oxidation/re-reduction of Au foils,^20^ also promoted by O_2_ plasma treatments,^31^ (ii) electroplating onto host templates,^32^ (iii) optimized electrodeposition^24^ or electrocrystallization with MHz potential oscillation,^33^ (iv) electron beam deposition,^25,26,34^ and (v) deposition of preformed Au nanostructures on conductive electrodes.^22,35,36^ In this context, straightforward one-step synthesis of porous Au structures with easily tunable morphology (upon appropriately changing the process parameters and not involving substrate limitations or thermal treatments) appears to be intriguing. These conditions could be fulfilled by pulsed-laser deposition (PLD), a highly versatile technique for the production of nanostructured films^37^ or nanoparticles^38^ of virtually any material, including metals,^39^ alloys,^40^ semiconductor oxides,^41^ and carbon.^42^ Highly porous structures are typically achieved by performing laser ablation in the presence of a background gas, and the resulting morphology can be easily tuned by controlling the gas pressure and/or target-to-substrate distance.^41,43,44^ Recently, some of us also showed that PLD can be used to produce Au nanoparticles with a precise control of size and substrate coverage while reporting their integration in the nanostructured TiO_2_ film by single-step deposition.^39,41^

In this contribution, we report on the pulsed-laser deposition of two different kinds of porous Au-nanostructured thin films on fluorine-doped tin oxide (FTO) electrodes and their use as cathodes for CO_2_ reduction in aqueous electrolytes. The accurate tuning of the deposition parameters allowed for the one-step synthesis of two nanoscale morphologies, one with a quite regular columnar arrangement and the other displaying a foamy tridimensional structure. The two nanoporous catalysts enabled the formation of syngas (CO + H_2_) mixtures of different compositions, together with small amounts of formic acid, both outperforming a planar gold foil used as a reference. Manifold setups and technological solutions for the electrochemical syngas preparation have been reported to date.^45−49^ Among them, the electrochemical generation of syngas mixtures at low overpotentials suits well in a CO_2_ valorization scenario, especially considering that one of the major costs in the whole Fischer–Tropsch processes is the syngas production itself (usually originating from methane or coal *via* steam reformation^50^).

## 2 Experimental Section

### 2.1 Materials

TEC 8 (8 Ω/sq) fluorine-doped tin oxide (FTO) conductive glass slides were purchased from Pilkington. FTO slides were cleaned by 10 min sonication in an Alconox aqueous solution, followed by 10 min sonication in 2-propanol. Gold foil (0.05 mm thick, 99.95%) and Nafion N-117 membrane (0.180 mm thick) were purchased from Alfa Aesar. Gold foils were cleaned according to literature procedures.^20^ Cr grains (99.99%) were purchased from Ista (Faenza), while CO_2_ (>99.9%) was from SOL Group. CO (99.0+%), NaOH (98%), 2-propanol, Alconox, and spectroscopic-grade acetonitrile were purchased from Sigma Aldrich. KHCO_3_ (99.5%) and KPF_6_ (>98%) were respectively purchased from Riedel-De Haen and Fluka, while Pb(NO_3_)_2_ (99%) was purchased from Carlo Erba. Unless otherwise stated, all chemicals were used without additional purification. All electrolytic solutions were prepared using reagent-grade water (Millipore, 18 MΩ·cm resistivity).

### 2.2 Cathode Preparation and Structural/Morphological Characterization

The cathodes consist in Au nanoporous films deposited on FTO substrates covered by a Cr adhesion layer that is needed to avoid the detachment of the Au deposit during the electrochemical tests. The 5 nm-thick Cr layer was deposited on FTO substrates in an Edwards E306 thermal evaporator by evaporating pure 99.99% Cr grains, while the equivalent thickness was controlled by means of a quartz microbalance. Au nanoporous films were then deposited on FTO substrates covered by the Cr interlayer via pulsed-laser deposition (PLD). A Au (99.99%) target was ablated with a nanosecond-pulsed laser (Nd:YAG, second harmonic, λ = 532 nm, repetition rate of 10 Hz, pulse duration of 5–7 ns); the laser fluence on the target was 2.3 J/cm^2^, while the laser pulse energy was 150 mJ. The substrates were mounted on a rotating sample holder at a fixed target-to-substrate distance of 5 cm. Depositions were performed at room temperature within a pure Ar background gas at two different pressures, 100 and 1000 Pa, for a duration of 20 min (12,000 shots). To distinguish Au nanoporous cathodes deposited at different Ar pressures, we name the films deposited at 100 and 1000 Pa as Col-Au and Foam-Au, respectively, as a result of their different morphologies (*vide infra*). Both films were deposited also on Si(100) substrates and added to the sample holder together with FTO substrates for the purpose of film characterizations. These films were compared to the Au foil as a reference cathode with a flat surface to evaluate the effect of the two different nanostructures obtained by PLD.

A field emission scanning electron microscope (Zeiss Supra 40) was used to perform morphological characterization on the films deposited on both Si and FTO substrates. In particular, the Si substrates were exploited for cross-sectional and top-view measurements, while the films deposited on FTO were scanned only on top view. Moreover, the scanning electron micrographs were analyzed by ImageJ software to extract the substrate coverage and size of morphological features of different Au films.

Structural characterization of deposited Au films was carried out by X-ray diffraction (XRD). XRD patterns were collected using a high-resolution X-ray powder diffractometer (PANalytical X’Pert Pro MPD) using a Cu target (CuKα1 radiation, −1.5406 Å) at room temperature. The measurements were performed in Bragg–Brentano (θ–θ) geometry with a step-scan technique in a 2θ range of 25°–85° with a step size of 0.016° and a time step of 40 s. The Bragg–Brentano geometry implies that X-ray diffraction occurs in the crystallographic planes that are parallel to the substrate; thus, XRD peak intensities can provide information about the presence of preferential orientation of crystalline domains with respect to the substrate. The size of the Au crystalline domains was determined by using Scherrer’s equation on XRD fitted peaks.

Transmission electron microscopy (TEM) images were obtained with a TEM JEOL 2010 with a LaB_6_ emission gun operating at 160 kV. High-resolution images, energy dispersive X-ray spectroscopy (EDS), and scanning transmission electron microscopy high-angle annular dark-field imaging (STEM-HAADF) analysis were performed with a FEI Titan HRTEM microscope operating at 80 kV. The Au samples were scratched from the Si substrate and deposited on copper TEM grids.

X-ray photoelectron spectroscopy (XPS) measurements were performed with a PHI 5000 VersaProbe II XPS System (Physical Electronics) with a monochromatic AlKα source (15 kV, 50 W) and photon energy of 1486.7 eV. The spectra were evaluated with MultiPak (ULVAC-PHI, Inc.) software.

### 2.3 Electrolyte Purification

As widely reported, the presence of metal cation impurities (especially Fe^2+^, Pb^2+^, and Zn^2+^) in the electrolytic solutions used for the CO_2_ electroreduction can result in unreliable results.^3^ Indeed, under the cathodic conditions needed for the reaction to proceed, these metal cations can be reduced to the corresponding metals and deposited onto the cathodic surface, leading to a significant modification of its catalytic properties. In particular, in the presence of these codeposited metals, the overpotential for proton reduction is reduced, leading to enhanced H_2_ production over CO_2_ reduction. Although nanostructured electrodes are less sensitive to this poisoning,^23^ metal impurities were removed by pre-electrolyzing the electrolytic solution using two large-area titanium foils kept at −2 V under nitrogen bubbling for 15 h.^51^ The effectiveness of the pre-electrolysis process has been proven by ICP-mass analysis, evidencing the absence of Fe^2+^, Pb^2+^, and Zn^2+^ in the limits of technique sensitivity (<0.5 ppm).

### 2.4 Electrochemical Measurements

#### 2.4.1 Determination of ECSA (Electrochemical Surface Area) by Double-Layer Capacitance Measurements

Experiments were carried out using an Autolab PGSTAT30 potentiostat in a three-electrode setup using a Pt foil as the counter electrode and a saturated calomel electrode (SCE) bathed in a saturated KNO_3_ solution as the reference. The electrolyte was prepared by dissolving 0.1 M KPF_6_ in acetonitrile. The CV sampling mode was set to “normal linear scan”, thus allowing for a true analog linear sweep instead of the incremental potential steps of typical digitalized potentiostats (staircase mode). CV scans for Au foil, Col-Au, and Foam-Au were recorded at scan rates in the range of 5–50 mV/s, spanning ±40 mV of the OCP, a range where no faradic processes occur. The current values were divided by the geometric area of the electrodes, which was determined using a stereomicroscope (OPTIKA, at 10× magnification) with a millimeter-sized transparent grid. From the CV traces, the capacitive current was then calculated as (*J*_a_ – *J*_c_)/2, where *J*_a_ and *J*_c_ are, respectively, the anodic and cathodic current densities at OCP. The resulting values (in A/cm^2^) were plotted against the scan rate of the CV experiments (in V/s) and the data fitted with a linear equation. The slope of the linear regression gives the capacitance of the electrode (in F/cm^2^). Assuming the Au foil to be featureless (roughness factor, RF = 1 by definition), the RFs of Col-Au and Foam-Au electrodes can be calculated by dividing the corresponding capacitance values by the capacitance of the Au foil used as the reference.

For each cathode, the OCP value was directly read on the potentiostat display after connecting all the three electrodes. The reading was stable. The OCP values for the different electrodes do not differ significantly in day-to-day use, with maximum variations within 70 mV. The ohmic resistance values, measured by electrochemical impedance spectroscopy, are in the range of 14–16 Ω for Col-Au and Foam-Au samples, while 6–10 Ω were obtained for the Au foil.

#### 2.4.2 Determination of Surface-Exposed Crystallographic Facets: Pb Underpotential Deposition

Experiments were carried out in a three-electrode setup using a Pt foil as the counter electrode and a saturated calomel electrode (SCE) as the reference. The electrolyte was prepared by dissolving 1 mM Pb(NO_3_)_2_ in 0.1 M NaOH and then purging with N_2_ prior to CV scans.

#### 2.4.3 Determination of Bridged CO (CO Stripping)

The surface coverage of CO molecules, kinetically inert and irreversibly bound to the nanoporous Au cathode (indicated in the main paper as CO_bridge_ species), can be estimated using the method described by Surendranath’s group.^52^ Briefly, the stripping cycles consisted in three successive linear scans: the first scan (up to 0.75 V vs SCE) allows for the registration of the oxidation peak due to the bielectronic stripping of the CO_bridge_ species; the second one starts at 0.75 V and stops at −0.14 V vs SCE since scanning to more negative values would restore CO_bridge_ species;^52^ and the third one, from −0.14 V back to 0.75 V vs SCE, serves as the baseline for the integration of the first linear scan to quantify the CO stripping charge after correcting for the scan rate (0.05 V/s). The stripping cycles were recorded immediately after the bulk electrolyses for the accumulation of the products.

#### 2.4.4 Product Accumulation and Analysis

Carbon dioxide electroreduction experiments were carried out in a modular custom-made polymethylmetacrylate (PMMA) cell. An ion exchange membrane (Nafion 117) divided the cell into two separated compartments. In the cathodic one, the working (Au-based cathodes) and reference (SCE) electrodes were located, while in the anodic compartment, the Pt counter electrode was immersed. Both the anolyte and catholyte consisted in a pre-electrolyzed 0.5 M KHCO_3_ aqueous solution, saturated with CO_2_ (resulting pH = 7.4). The working electrodes were electrically connected to Cu wires using silver paint, and then epoxy resin was used to isolate every part but the catalytic surface. We did not extend the scans to potentials lower than −0.62 V vs RHE since deterioration of the FTO substrates under exceedingly cathodic conditions can occur.

Stepped chronoamperometric experiments have been performed to accumulate the products. In particular, 270 s at the fixed cathodic bias needed for the reduction reaction is followed by 30 s at an open-circuit potential to desorb the terminally bonded CO (CO_term_ in the main text) from the electrodic surfaces. For the sake of comparison with the majority of the literature, all the potential values applied in the CO_2_ reduction experiments have been reported also versus the reversible hydrogen electrode (RHE) using the formula
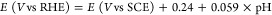


Unless otherwise stated, all the potential values concerning the CO_2_ reduction experiments are given vs RHE in the text, while the figures report also a second potential axis, with values referred to the saturated calomel electrode (SCE).

The cathodic compartment of the cell was connected to a headspace, from which the GC pump automatically collected samples for gas detection and quantification. The latter was performed by means of an Agilent Technologies 490 microGC equipped with a 5 Å molecular sieve column (10 m) and thermal conductivity detector, using Ar as the carrier gas. Fifteen milliliters from the headspace was sampled by the internal GC pump and 9 μL was injected in the column that is maintained at 90 °C. The uninjected gas was then reintroduced in the cell to minimize its variation along the whole experiment.

Hydrogen was quantified using a response factor obtained through galvanostatic electrolysis (10 mA, 1 h) of a 0.1 M H_2_SO_4_ solution in the same electrochemical cell, using a Pt working electrode and assuming 100% faradic efficiency of proton reduction. Carbon monoxide was quantified using a response factor obtained by injecting known amounts of CO in the electrochemical cell and then sampling the headspace. Quantification of formate was performed via ^1^H-NMR spectroscopy (Agilent, 300 MHz). At the end of the pulsed-bias chronoamperometry experiments at the specific potential, the catholyte was sampled and known amounts of DMF and D_2_O were added respectively as the external standard and locking solvent. The ^1^H-NMR spectrum was acquired using a customized water suppression sequence, allowing for the minimization of the aqueous electrolyte signal. Formate was easily identified as the singlet peak at 8.3 ppm and quantified by comparative integration with the DMF peaks.

For all the products, the faradic efficiency at the different applied biases could be calculated as follows
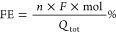
with mol being the amount of product (determined as described above), *n* being the number of electrons involved in the reduction reaction, *F* being the Faraday constant, and *Q*_tot_ being the total amount of charge passed at the interface during the pulsed bulk electrolysis experiments, obtained from the integration of the chronoamperometric curve over time.

## 3 Results and Discussion

### 3.1 Synthesis and Characterization of the Au Nanostructures

**Figure 1:**
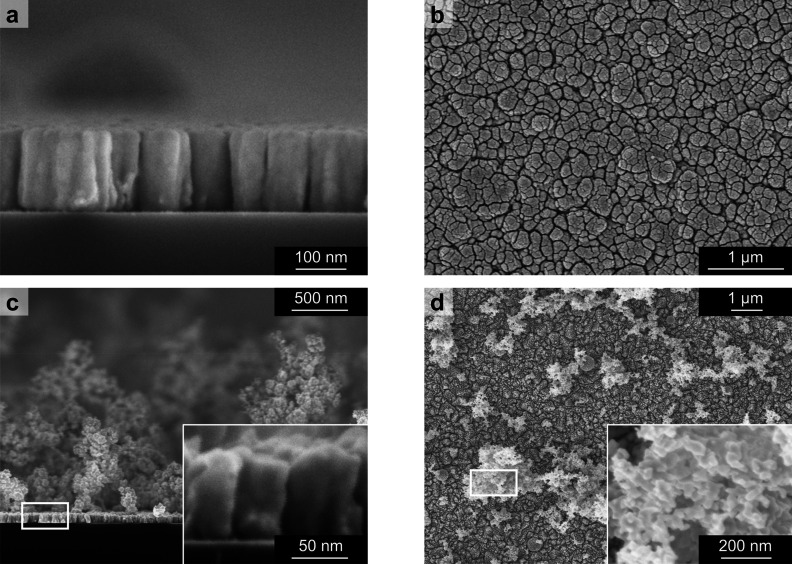
SEM images (top view and cross section) of Au films: (a, b) Col-Au deposited at 100 Pa and (c, d) Foam-Au deposited at 1000 Pa. Insets in (c) and (d) show images at higher magnification.

The nanostructured Au cathodes were deposited by means of pulsed-laser deposition (PLD) on FTO substrates covered with a thin (5 nm) Cr adhesion layer prepared by thermal evaporation. Figure 1 shows the cross-sectional and top-view scanning electron microscopy (SEM) images of Au films deposited at 100 and 1000 Pa of Ar, highlighting their different morphologies as a function of background deposition pressure. Indeed, the Au film deposited at 100 Pa shows a columnar structure for its whole thickness (Figure 1a,b); on the other hand, the Au film deposited at 1000 Pa exhibits a columnar-like structure only for a bottom ∼80 nm-thick layer in contact with the substrate, while the main structure consists of a non-uniform foam-like morphology up to a few micrometers in thickness (Figure 1c,d). For these morphological features, the following 100 Pa- and 1000 Pa-deposited films are called Col-Au and Foam-Au, respectively. In particular, the Col-Au film consists of ∼200 nm-thick and ∼80 nm-wide columns (Figure 1a), on average, separated by voids of the order of 10–15 nm (Figure 1b). On the other hand, the Foam-Au film shows a column-like bottom layer, sizing about 80 nm thick and 45 nm wide (Figure 1c), also separated by voids of the order of 10–15 nm (Figure 1d). Moreover, the foam-like structure on top is up to 3–4 μm thick and appears to be composed of sintered Au nanoparticles with a size of a few tens of nanometers (average size, 35 nm). Such foam-like domains cover ∼20% of the substrate surface (Figure 1d).

The evident difference in morphology as a function of the background pressure is due to the coexistence of two different mechanisms of film growth during deposition, namely, in-plume cluster nucleation and surface diffusion.^53,54^ When other PLD parameters (e.g., laser energy and fluence and target-to-substrate distance) are kept constant, the predominance of one mechanism over the other is associated to the pressure level.^39^ Indeed, during the PLD process, the laser–target interaction leads to target vaporization, which results in plasma plume formation and consequent deposition of ablated species on the substrates.^38,43,55^ The increment in background pressure from 100 to 1000 Pa has the effect of confining the plasma plume more effectively as well as slowing down the ablated species. Therefore, in-plume cluster nucleation phenomena are more predominant at 1000 Pa rather than at 100 Pa, resulting in the deposition of a more open and fluffier Au nanoporous film with the different morphologies already described. The presence of the “compact” columnar bottom layer for the Foam-Au cathode deposited at 1000 Pa is probably related to the initial wetting of the substrate by means of the ablated Au.

Moreover, the background pressure level also affects the deposition rate as the higher pressure means stronger scattering and thus a less directional ablation plume, which translates in higher dispersion within the deposition chamber and lower kinetic energy. The amount (mass density per unit surface) of Au deposited at the two pressure conditions estimated by means of a quartz microbalance was ∼300 μg/cm^2^ for Col-Au and ∼150 μg/cm^2^ for Foam-Au. The deposition of such small amounts of gold is indeed advantageous for the overall cost of the cathodes.

**Figure 2:**
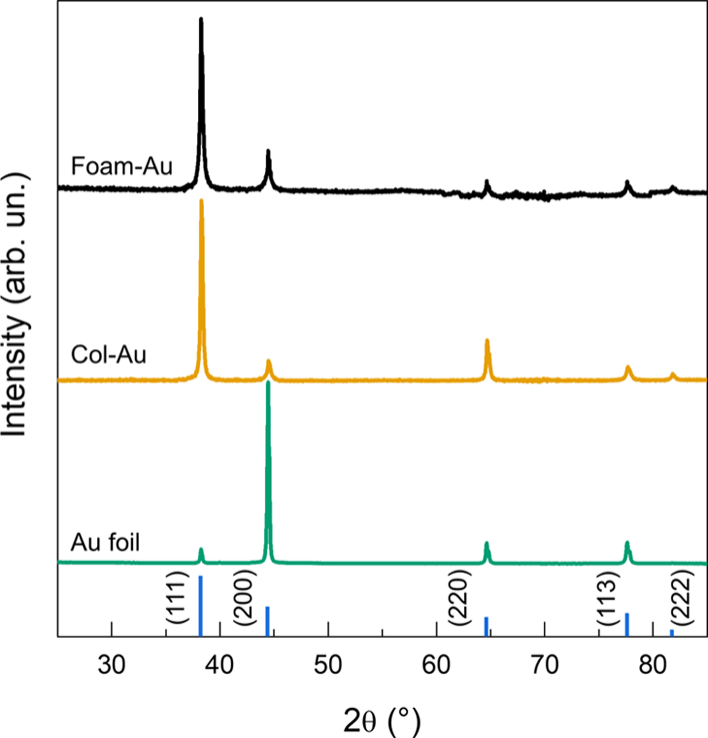
X-ray diffractograms of Col-Au, Foam-Au, and Au foil; intensities have been normalized to the (111) reflection (Col-Au and Foam-Au) and (200) reflection (Au foil). The characteristic peaks of the Au fcc structure in a powder system are reported as the reference (PDF database card no. 00-004-0078); the height of the reference lines is proportional to the intensity of XRD reflections in reference Au powder.

The structural characterization of Au samples was performed by means of X-ray diffraction (Figure 2). Specifically, both the Col-Au and Foam-Au films show peak positions in accordance with the Au fcc structure; the higher signal-to-noise ratio of Col-Au indicates better crystallinity for this film. The relative intensities of XRD peaks differ for both samples from those of reference Au powder with random orientation of crystalline domains. This is a clear indication of preferential crystalline domain growth with respect to the substrate. In detail, both films preferentially grow along the (111) direction; furthermore, for Col-Au, growth along the (220) direction also appears to be preferred with respect to the (200) one. On the other hand, the Au foil exhibits preferential orientation along the (200) direction. The average size of Au crystalline domains was estimated by applying Scherrer’s equation on the Au (111) fitted peak, resulting in 37 and 29 nm for Col-Au and Foam-Au, respectively.

**Figure 3:**
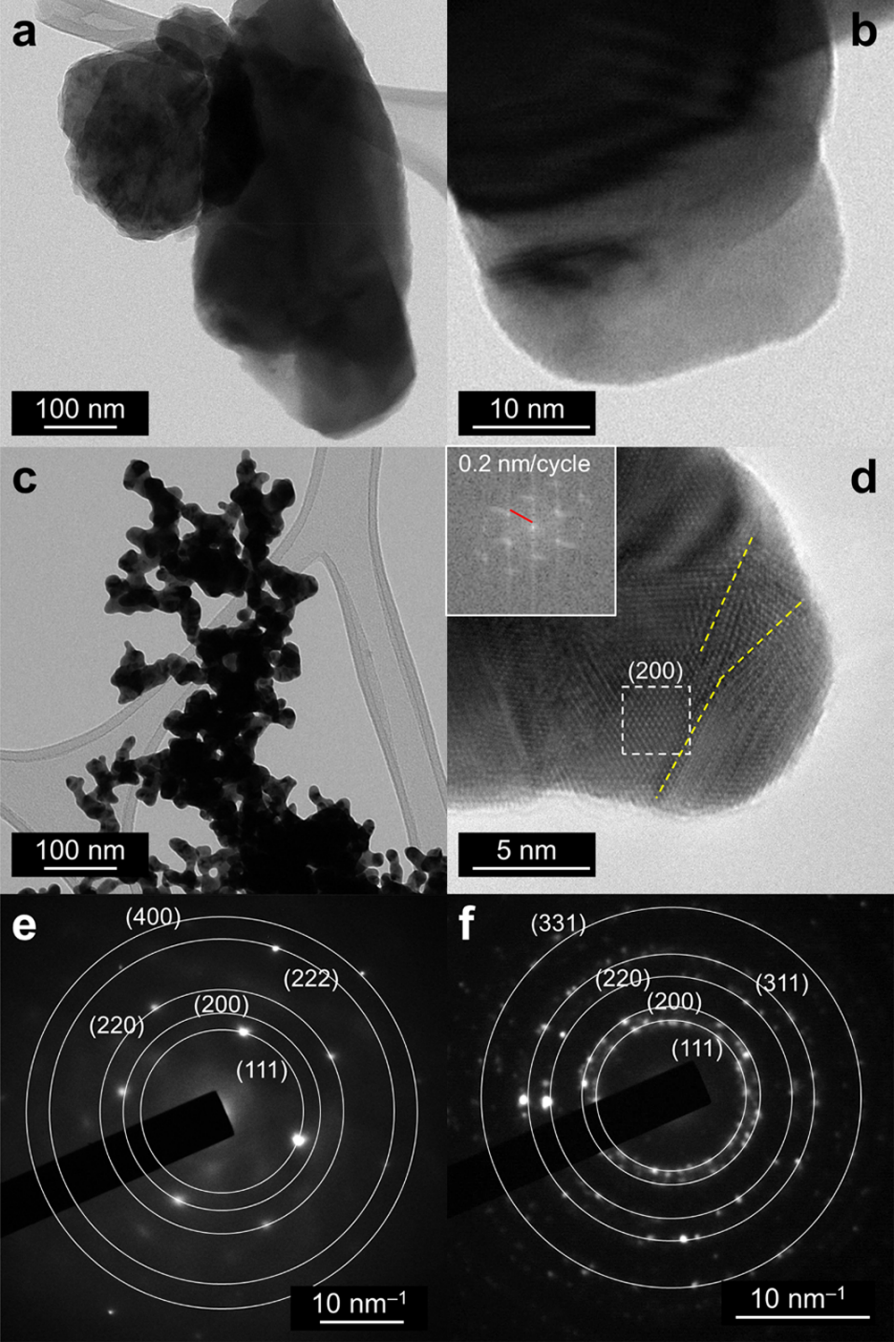
TEM images of (a, b) Col-Au and (c, d) Foam-Au. The inset in (d) shows the Fourier transform of the atomically resolved area highlighted by a dashed box; the yellow dashed lines mark grain boundaries. (e, f) Selected area electron diffraction (SAED) of the micrograph reported in Figure S1a,b, respectively, for Col-Au and Foam-Au, showing diffraction spots corresponding to the lattice planes of pure Au (camera length values of 2.25 and 1.30 were respectively used to correct the interplanar distance values).

TEM analysis was performed to gain more insights into the local structural properties of the nanostructured Au films (Figure 3). Figure 3a shows a portion of the Col-Au film, which appears to be dark due to its high density, thus preventing the acquisition of atomically resolved images (Figure 3b). Figure 3c shows the foam-like structures growing on top of the Foam-Au film, while Figure 3d is a high-resolution TEM image with atomic resolution. In this case, grain boundaries could be discerned (yellow dashed lines in Figure 3d) as well as (200) planes on the surface. This observation suggests the presence of randomly oriented grains in the Foam-Au film. We anticipate that the presence of (200) facets can lead to relevant effects in terms of the faradic efficiency toward CO_2_ reduction to CO (see below).^29^ Indeed, by analyzing larger areas with TEM to acquire SAED patterns (Figures S1a and 3e for Col-Au and Figures S1b and 3f for Foam-Au), a larger number of diffraction spots were found for the Foam-Au sample, thus confirming the above observation. Finally, the high level of purity of the Au nanostructured films was confirmed by energy dispersive X-ray spectroscopy (EDS) mapping (see the STEM-high-angle angular dark-field (HAADF) micrograph in Figure S1c and the corresponding EDS map in Figure S1d).

The Col-Au and Foam-Au cathodes were initially characterized through different electrochemical techniques that were able to provide insights into both their active area and the exposed facets. As regard to the first aspect, double-layer capacitance (DLC) measurements are widely used^56−61^ since they represent a versatile nondestructive technique to estimate the electrochemical surface area (ECSA). However, it is worth noting that several processes involving ion transfer reactions at the interface (e.g. intercalation, specific adsorption, or surface proton transfer) can lead to additional contributions altering the actual capacitance values, especially in aqueous media. Thus, we performed the DLC experiments in acetonitrile, a polar aprotic solvent in which more uniform capacitance values for different materials can be obtained, following a recent procedure reported by Surendranath’s group.^62^ From the analysis of the cyclic voltammetries at different scan rates reported in Figure S2, compared to the ones obtained for a flat Au foil, we could estimate roughness factor values of 12.7 ± 3.1 for Col-Au and 9.1 ± 1.0 for Foam-Au (see also Table S1), most likely reflecting the trade-off between the opposite contributions due to the different morphologies of the cathodes and their total gold loading. The values confirmed the high porosity of both the nanostructures and were in line with roughness factors reported for cathodes with comparable morphologies.^34^

**Figure 4:**
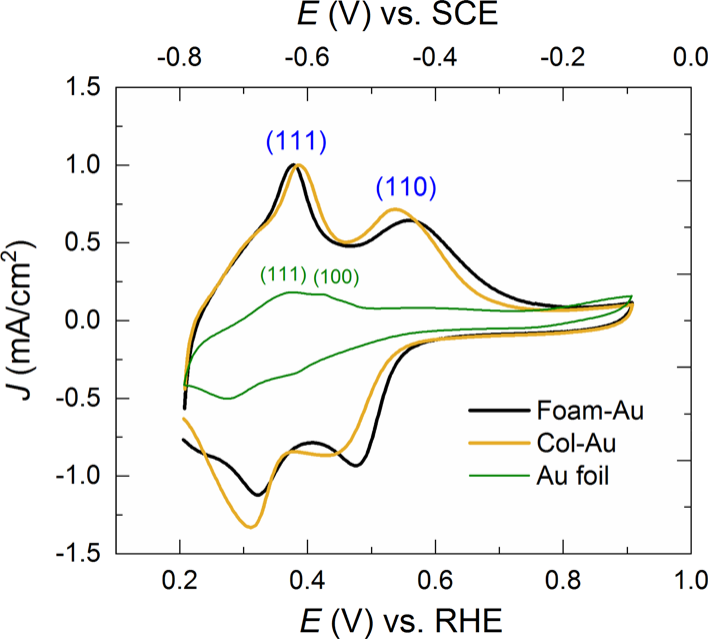
Pb UPD traces recorded in 1 mM Pb(NO_3_)_2_ + 0.1 M NaOH with a 25 mV/s scan rate. (111) and (110) facet orientations are revealed at 0.35 and 0.50 V, respectively, while (100) is at 0.40 V. The curves for Foam-Au and Col-Au were normalized to match the peak heights of the (111) feature.

To gain insights into the distribution of the Au surface terminations of the two nanoporous structures, underpotential deposition (UPD) of Pb was performed. Results are reported in Figure 4, where the two reversible processes at *E*_1/2_ = 0.35 and 0.50 V vs RHE respectively correspond to Pb deposition and stripping from the (111) and (110) exposed facets of the Au cathodes, in agreement with XRD analyses (the (100) facet could be revealed at 0.40 V only for Au foil, reported as a reference).^26,32,63,64^ For both Col-Au and Foam-Au, the relative amplitude of each wave was quite similar, suggesting negligible dependence of the Au surface termination on the morphology of the cathodes. Similar behavior was previously observed in Au-inverse opal thin films^32^ as well as on carbon nanotubes decorated with Au nanoparticles deposited via e-beam evaporation.^26^ Anyway, in both Col-Au and Foam-Au morphologies, the density of the (111) facets is higher with respect to that of (110). These results can translate in reduced selectivity for the CO_2_ versus the proton reduction reaction since the more opened and undercoordinated (110) sites have been recently reported to be *ca.* 6 times more active for CO production than the (111) sites.^29^

### 3.2 Electrochemical Performances of the Au Nanostructures

The so-prepared cathodes were tested as working electrodes in a custom-made electrochemical cell (see Section 2 and Figure S3 for a more detailed description of the experimental setup) using pre-electrolyzed 0.5 M KHCO_3_ saturated with CO_2_ as the electrolytic solution. The joined presence of these two species leads to the formation of a buffer system at pH 7.4, instrumental to avoid the buildup of a basic pH (and the consequent decrease of the dissolved CO_2_) following proton consumption during electroreduction.

The cations of the electrolyte (K^+^ in this specific case) are also known to participate in the buffering process since their hydration shell can be polarized and then undergo hydrolysis under cathodic biases.^23,65,66^ Furthermore, the K^+^ absorbed on the electrodic surface may favor the stabilization of the intermediate anionic species via ion pairing^3,23,67^ and, at the same time, by decreasing the competitive H_2_ evolving reaction due to the buildup of a more positive potential in the Helmholtz layer.^23,68^

**Figure 5:**
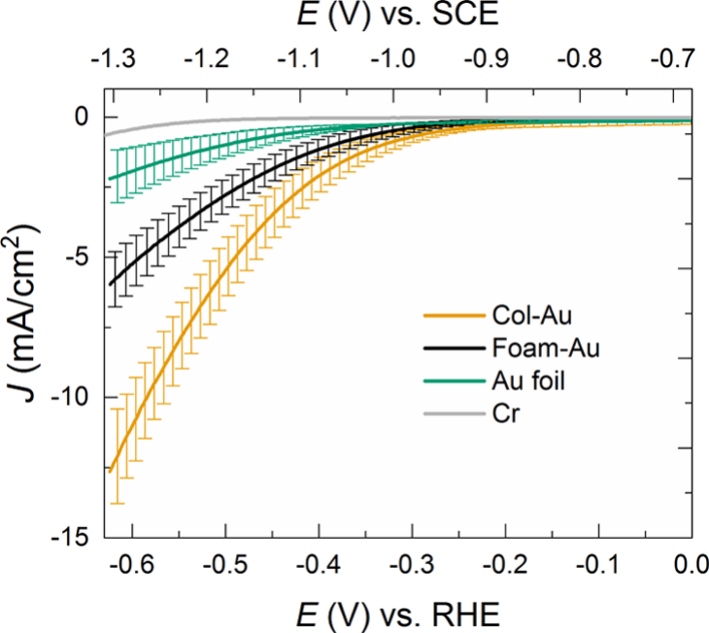
*J*–*E* characteristics for Col-Au (dark yellow), Foam-Au (black), Au foil (green), and Cr adhesion layer (gray) recorded in 0.5 M KHCO_3_ saturated with CO_2_ (pH 7.4), normalized for the geometric area and corrected for the iR drop. The stable response (i.e., the second one of successive *J*–*E* cycles) of at least three electrodes has been averaged, and the corresponding curves are reported, together with the error bars.

Figure 5 shows the resulting *J*–*E* curves recorded at 10 mV/s while compensating for the ohmic drop. All the traces correspond to average values of at least three equivalent electrodes, and the corresponding standard deviations are also reported as error bars, evidencing the good reproducibility of the outcomes in terms of generated current. The performances of the two nanoporous cathodes were also compared to those of a commercial Au foil as the standard reference as well as to those of the bare Cr adhesion layer (*J*–*E* curves normalized for the ECSA are reported in Figure S4).

The analysis of the *J*–*E* curves revealed that the onset potential at which a significant cathodic current (in the specific, −0.5 mA/cm^2^) starts to flow is −0.27 V for Col-Au, corresponding to an overpotential |η| = 0.16 V, with −0.11 V being the thermodynamic potential for CO_2_ reduction to CO. This onset value is pretty similar to the one registered for the pioneering oxide-derived Au nanostructures reported by Kanan’s group.^20^ Less negative onsets (−0.2 V) were however observed for very peculiar Au nanostructures, such as Au needles, for which high local electric fields arise, resulting in a higher local CO_2_ concentration.^24^

On the other hand, the onset potential for Foam-Au was observed at −0.32 V (|η| = 0.21 V), with *ca.* −0.05 V cathodic onset shift with respect to Col-Au. This shift can be due to subtle differences in the reaction kinetics at the two different interfaces, which can translate into different product distributions (*vide infra*). At the same time, the two nanoporous morphologies outperformed the Au foil, for which the current onset is observed at −0.42 V (|η| = 0.31 V), thus speaking in favor of improved kinetics in the nanostructured interfaces when compared to the flat Au surface. The Cr adhesion layer showed, as expected, a very retarded onset potential (at −0.61 V, |η| = 0.5 V) with the recorded current being essentially due to hydrogen generation.^3^

The catalytic activity, in terms of generated current, follows the trend Au foil < Foam-Au < Col-Au, with the latter reaching up to −12.5 mA/cm^2^ at −0.62 V. However, the ultimate assessment of the catalytic performances of the nanoporous cathodes must be done after the evaluation and quantification of the reduction products.

To this end, we performed chronoamperometric measurements under different potentials (Figure S5) since the distribution of CO_2_ reduction products is known to change upon varying the applied bias. However, while performing these experiments, we noticed a progressive decrease in the cathodic currents, which we attributed to partial poisoning of the cathodic surfaces. This behavior had been already reported for Au surfaces and ascribed to different kinds of adsorbed species, either potassium and/or carbon deposits^69^ or the produced CO itself.^52^ In particular, terminally bonded CO species (CO_term_) have been reported to be only reversibly absorbed on the Au surface (lowering, however, the fraction of sites available for the catalysis), so they can be easily removed under open-circuit potential (OCP) conditions. Indeed, when the chronoamperometric protocol was modified, introducing short reconditioning steps at OCP, the initial current density values for both Foam-Au and Col-Au were restored (see Figure S5), allowing for the assessment of the medium term stability of the cathodes as well as for the accumulation of sufficient amounts of products for their quantification. It is worth noting that even if the reconditioning step can be considered as a “dead time” in the whole process, it accounts only for the 10% of the total electrolysis time (30 s every 270 s). The recovery of the initial current density values for the Foam-Au and Col-Au samples, moreover, suggested good mechanical stability during the electrochemical measurements, which was attributed to the Cr adhesion layer between Au and the FTO substrate.

**Figure 6:**
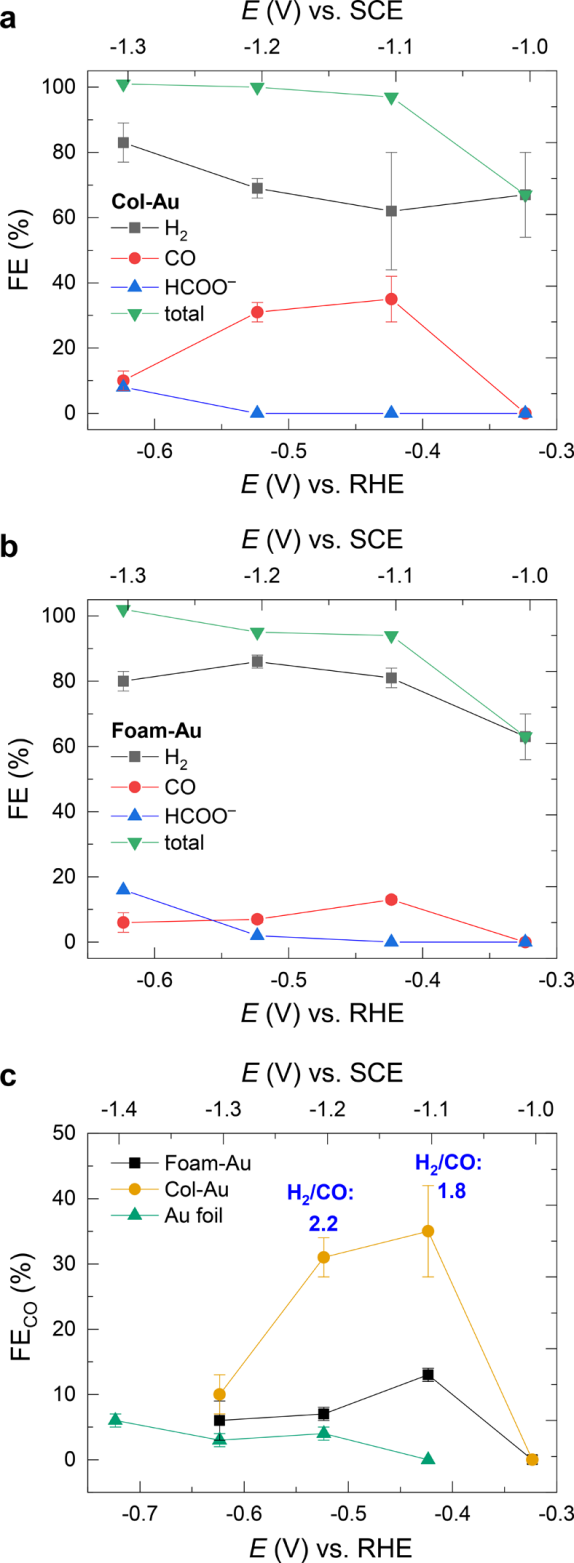
Faradic efficiency of the different products obtained with (a) Col-Au and (b) Foam-Au cathodes as a function of the applied bias. Each point is an average of at least three measurements, and the corresponding standard deviations are reported as error bars. The <100% total FE observed at −0.32 V (low current, i.e., lower amount of products) can be due to trapping of the gas products in stagnant corners of the electrochemical cell. (c) Applied bias dependence of the faradic efficiency for the generation of CO for the different cathodes.

The faradic efficiencies (FE) of the different products as a function of the applied bias are reported in Figure 6 and Table S2. Col-Au electrodes yielded CO as the major CO_2_ reduction product (up to 35% FE at the low |η| value of 0.31 V) as well as small amounts (<8%) of formic acid at −0.62 V (Figure 6a). At the same time, hydrogen also evolved as a consequence of the competing proton reduction. Anyway, H_2_/CO with a ratio of ∼2 registered at an intermediate bias (−0.42 and – 0.52 V) is a particularly appealing gas mixture, being compatible with important industrial processes, such as hydrocarbons or methanol production via Fischer–Tropsch syntheses.^13−15^

On the other hand, the Foam-Au surface more markedly favors proton over CO_2_ reduction (Figure 6b). Indeed, the maximum FE value for CO evolution was 13% at −0.42 V (|η| = 0.31 V), still overcoming the Au foil, for which <6% of carbon monoxide was observed in the whole investigated potential range (Figure 6c). The H_2_/CO ratio for Foam-Au is thus >2 (see Table S2 for further details), envisaging the use of these gaseous mixtures for the production of short-chain hydrocarbons (e.g., methane) via Fischer–Tropsch syntheses, with H_2_ being involved in chain termination processes.^15^ Furthermore, the syngas mixtures with higher hydrogen content could be used for biological fermentations since the specificity of the involved enzymatic reactions makes this kind of process less dependent on fixed H_2_/CO compositions. Indeed, syngas mixtures obtained from the pyrolysis of solid waste and organic residues were recently fed to microorganisms, yielding biodegradable plastics, such as polyhydroxyalkanoates (polyesters).^70,71^

When compared to other nanoporous structures reported in the literature, which usually show the selective formation of CO in aqueous media (FE > 95%),^18,20,24,25,32^ both our cathodes produced significantly higher amounts of H_2_, speaking in favor of preferential absorption of *H over *CO_2_ (likely as *COO^–^)^52^ on Col-Au and Foam-Au surfaces. A possible explanation of this behavior could be related to a low amount of grain boundaries in our morphologies, mostly present in the foam-like domains of the Foam-Au film (Figure 3d) rather than in the vertically oriented Col-Au (Figure 1a) and in the bottom layer of Foam-Au (Figure 1c). Indeed, the surface density of grain boundaries has been linearly correlated to CO_2_ reduction activity.^26^ On the other hand, the higher faradic efficiency in CO production of the Col-Au film with respect to the Foam-Au film (Figure 6a,b) could be related to a different relative abundance of undercoordinated facets, such as (110), with respect to facets with a high coordination number, such as (111) and (200). Indeed, XRD results (Figure 2) and TEM analysis (Figure 3) suggest a higher amount of (200) facets for the Foam-Au film. According to a recent report,^29^ the equivalent (100) facets exhibit a significantly lower faradic efficiency toward CO evolution rather than the undercoordinated (211) and (110). Consistently, the Col-Au film, which exhibits a stronger (220) diffraction peak, i.e., equivalent to the (110) lattice plane family, produced a higher amount of CO. Moreover, the Au foil, mostly exhibiting the highly coordinated (200) facets, produced very low amounts of CO (Figure 6c).

This aspect indirectly translates also in the different amounts of irreversibly bridge-bonded CO spectators (CO_bridge_) on the surface of the investigated cathodes. Indeed, using the oxidative stripping method described by Surendranath’s group,^52^ we could estimate the surface coverage of the CO_bridge_ spectators (see Figure S6, Section 2, and the Supporting Information for further details). Table S3 collects the calculated values, increasing in the order Au foil < Foam-Au < Col-Au, thus confirming the preferential coordination of CO on undercoordinated sites.

On the other hand, the FE for CO of the Au foil was ≤6% (Figure 6c), similar to previous studies^21,33^ but lower than in others, reporting FEs ranging from 10 to 40%^23,25,72^ but also up to more than 90%.^29^ The scattered FE_CO_ values reported in the literature may be in part due to molecular species adsorbed to the Au surface, which have shown the capability to regulate the selectivity of functionalized Au surfaces.^73^ To elucidate this aspect, XPS measurements were performed on the Col-Au and Foam-Au films (both before and after CO_2_ reduction experiments), as well as on the Au foil (Figure S7 and Table S4), evidencing only Au, C, and O peaks, thus ruling out any heterometallic contamination within the detection limits of XPS. Furthermore, in all the samples, the local chemical environment of the metallic Au surface was not affected by the electrochemical experiments (Figure S7). Substantial amounts of C and O in the form of oxyhydrocarbons^74^ were always detected , which were ∼40 and ∼10 atom %, respectively, for the nanostructured Au films, both for the pristine and tested samples, and ∼56 and ∼21 atom % for the Au foil (Table S4). Such high content of carbon detected on the Au foil surface^69^ may be responsible for its unusually low FE toward CO.^73^

Finally, it is worth noting that the performance of the Au electrodes could also be affected by parameters related to the experimental setup. In our case, the nanostructured Au cathodes likely experienced progressive local depletion of the gaseous substrate over time due to the limited mass transport of CO_2_, thus favoring H_2_ formation. This limitation may mask the beneficial effects arising from the buildup of local pH gradients, reported to inhibit H_2_ generation, thus enhancing the global CO_2_-to-fuel selectivity.^52^ To improve this aspect, we are currently optimizing a new custom-made electrochemical cell featuring a flow circulation of the electrolytic solution saturated with CO_2_.

## 4 Conclusions

We have successfully prepared two nanostructured porous Au cathodes through one-step synthesis using pulsed-laser deposition. By carefully tuning the deposition parameters, we could obtain high-porosity morphologies, displaying either a quite regular columnar arrangement or a foamy structure. When used as cathodes for the electrochemical reduction of CO_2_, the two electrodes displayed selective production of syngas mixtures of different compositions already at overpotentials as low as 0.31 V in aqueous media. In particular, with the Col-Au cathodes, we obtained quantitative conversion of charge into syngas (faradic efficiency) with a H_2_/CO ratio of ∼2, the most appropriate composition for Fischer–Tropsch processes aimed at the production of hydrocarbons or methanol. On the other hand, the Foam-Au cathodes produced syngas mixtures enriched in H_2_, which could be exploited either for the methane production or for biological fermentation to yield biodegradable plastics.

Starting from our results, one can envisage the design of reactors for the Fischer–Tropsch process or bioreactors directly fed by the gaseous mixture generated by our electrochemical cell, thus valorizing the waste gas CO_2_ while changing the paradigm of the concomitant production of H_2_ from a negative aspect to an asset.

## Supporting Information Available

TEM and STEM-HAADF images, EDS mapping, DLC measurements for ECSA determination, pictures of the setup, chronoamperometries and CO stripping analyses, and XPS analysis (PDF)
