# Supplementary material for: Syngas Evolution from CO2 Electroreduction by Porous Au Nanostructures
Source: ACS Appl Energy Mater. 2020 May 6;3(5):4658–68. doi: 10.1021/acsaem.0c00301 (PMC8016180; doi:10.1021/acsaem.0c00301)
Supplement: Supplementary file 1 — ae0c00301_si_001.pdf [file ae0c00301_si_001.pdf]

Supporting information to:

Syngas evolution from CO_2 electroreduction by porous Au nanostructures

Luca Mascaretti,[a,b] Alessandro Niorettini,[c] Beatrice Roberta Bricchi,[a] Matteo Ghidelli,[a,d] Alberto Naldoni,[b] Stefano Caramori,[c] Andrea Li Bassi,[a,*] Serena Berardi[c,*]

[a] Micro- and Nanostructured Materials Laboratory, Department of Energy, Politecnico di Milano, Via Ponzio 34/3, 20133 Milano, Italy

[b] Regional Centre of Advanced Technologies and Materials, Faculty of Science, Palacký University, Šlechtitelů 27, 78371 Olomouc, Czech Republic

[c] Department of Chemical and Pharmaceutical Sciences, University of Ferrara, Via Luigi Borsari 46, 44121 Ferrara, Italy

[d] Department of Structure and Nano/-Micromechanics of Materials, Max-Planck-Institut für Eisenforschung GmbH, Max-Planck Straße 1, 40237 Düsseldorf, Germany

[*] Corresponding authors: andrea.libassi@polimi.it; serena.berardi@unife.it

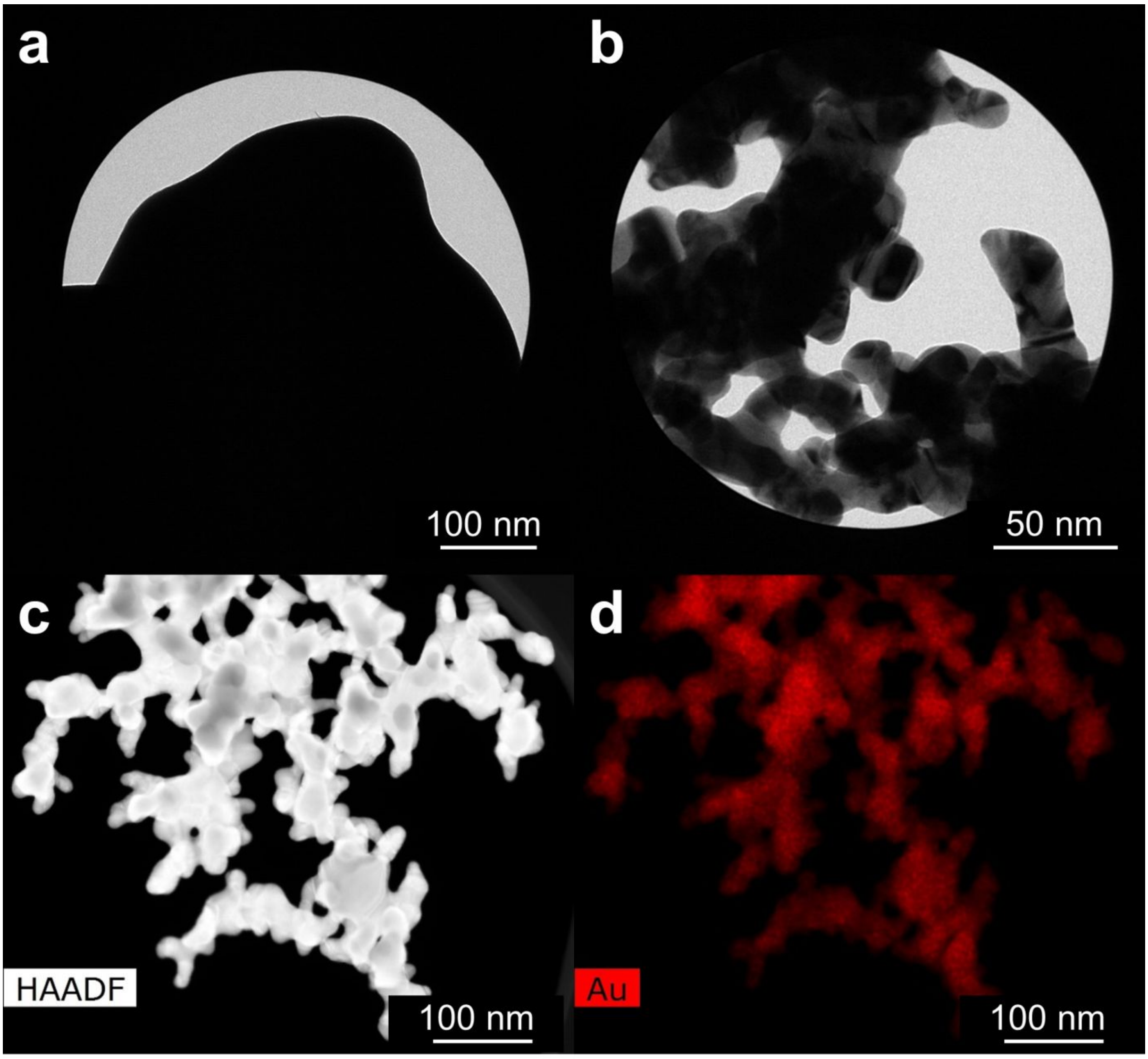

Figure S1. (a) TEM image of the Col-Au film used for the selected area electron diffraction (SAED) analysis reported in Figure 3e in the main text. **(b)** TEM image of the Foam-Au film used for the SAED analysis reported in Figure 3f in the main text. **(c)** STEM-HAADF (high-angle angular dark-field) image of Foam-Au. **(d)** Energy dispersive X-ray spectroscopy (EDS) mapping of the micrograph reported in **(c)**, showing the pure elemental composition of the Au nanoporous film.

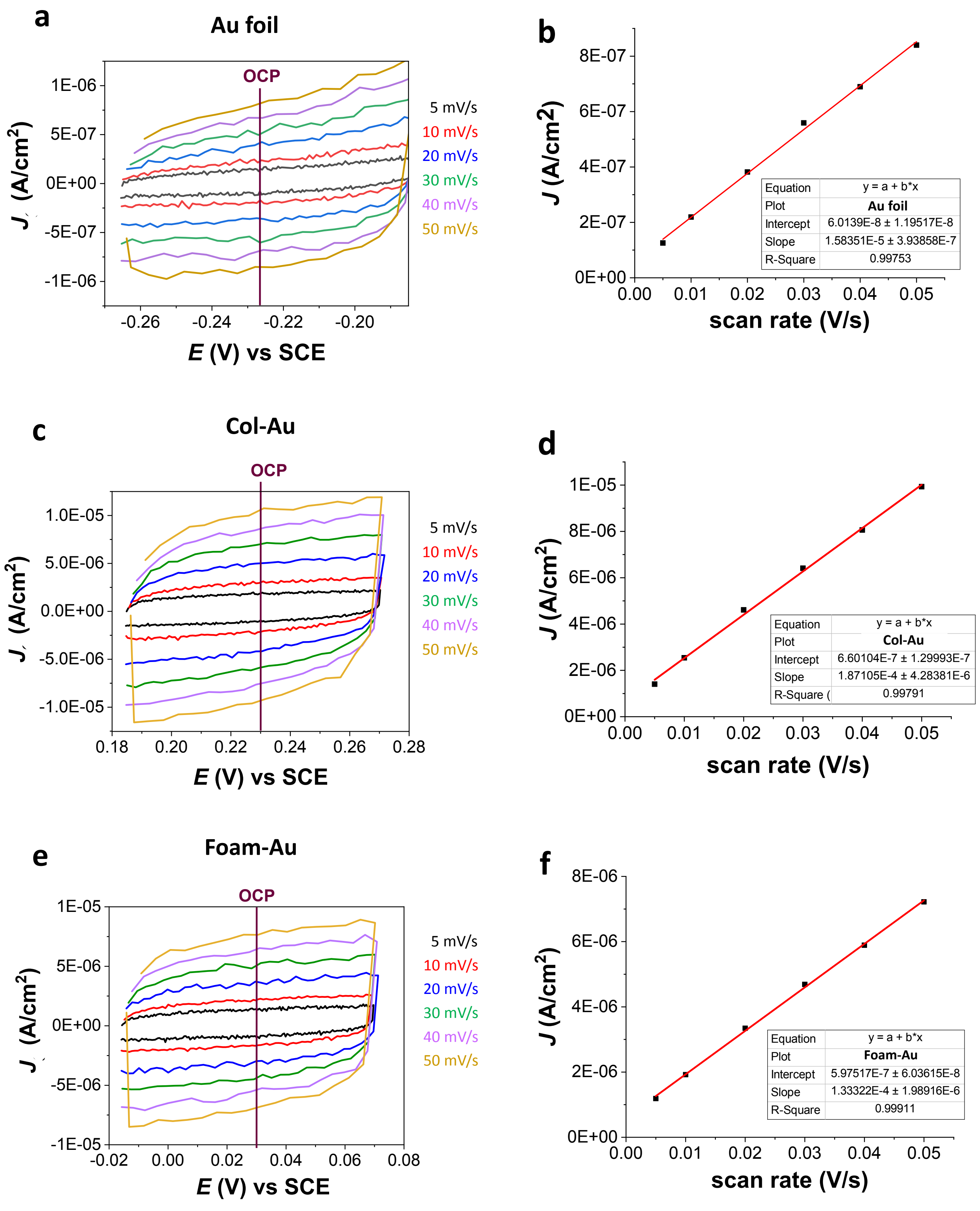

Figure S2. Double Layer Capacitance (DLC) measurements. CV scans around the open circuit potential (OCP) for Au foil **(a)**, Col-Au **(c)** and Foam-Au **(e)** registered in 0.1 M KPF_6 in acetonitrile. The corresponding linear fits are reported respectively in **(b)**, **(d)** and **(f)**. The calculated slopes correspond to the electrode capacitances, from which the RFs can be calculated as reported in the main paper. It is worth noting that these Figures are related just to one electrode of each kind, but up to 3 samples of each have been actually tested. The resulting average values are reported in Table S1.

Table S1. Capacitance values and surface roughness factors calculated from the DLC experiments reported in Figure S2. The surface roughness factor for the featureless Au foil used as the reference is defined to be 1. Average values for 3 Col-Au and 2 Foam-Au electrodes are reported .

	**Au foil**	**Col-Au**	**Foam-Au**
**Electrode Capacitance** (= slope of the linear fit)	$1.58 \cdot 10^{-5}$ **F/cm²**	$(2.01 \pm 0.49) \cdot 10^{-4}$ **F/cm²**	$(1.44 \pm 0.16) \cdot 10^{-4}$ **F/cm²**
**Roughness factor (RF)** (= Electrode Capacitance/ Au foil Capacitance)	1	12.7 ± 3.1	9.1 ± 1.0

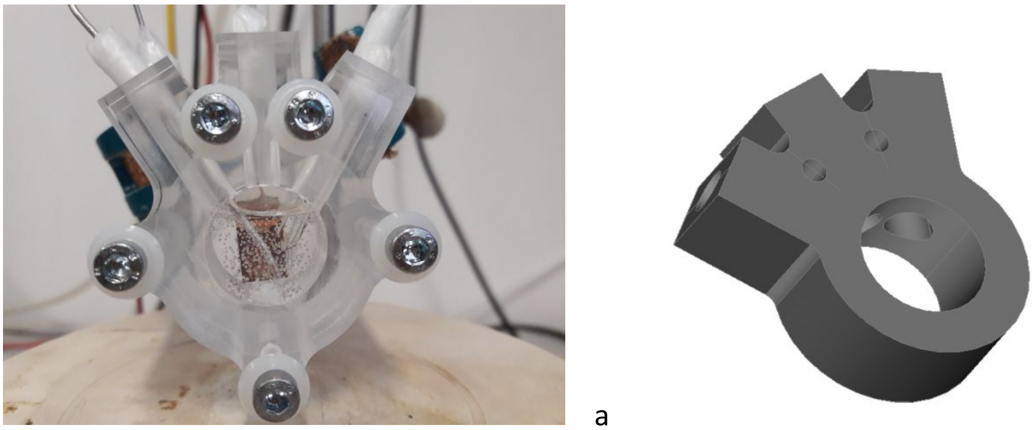
a b

Figure S3. Picture of the custom-made electrochemical cell used in this work **(a)** and 3D rendering of one of the modular components **(b)**.

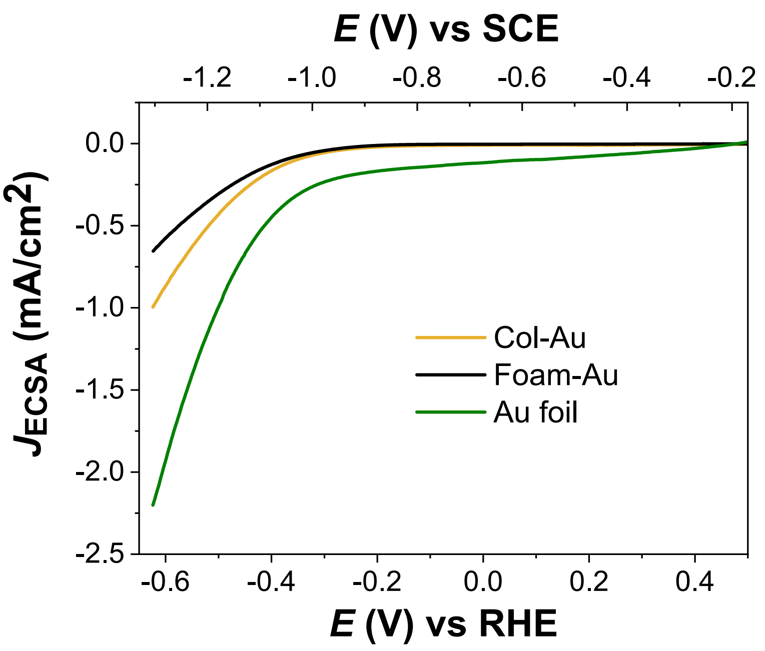

Figure S4. J-E characteristics for Col-Au (dark yellow), Foam-Au (black) and Au foil (green), all normalized for the corresponding ECSA values. The traces are recorded in 0.5 M $KHCO_3$ saturated with CO_2 (pH 7.4) and corrected for the iR-drop.

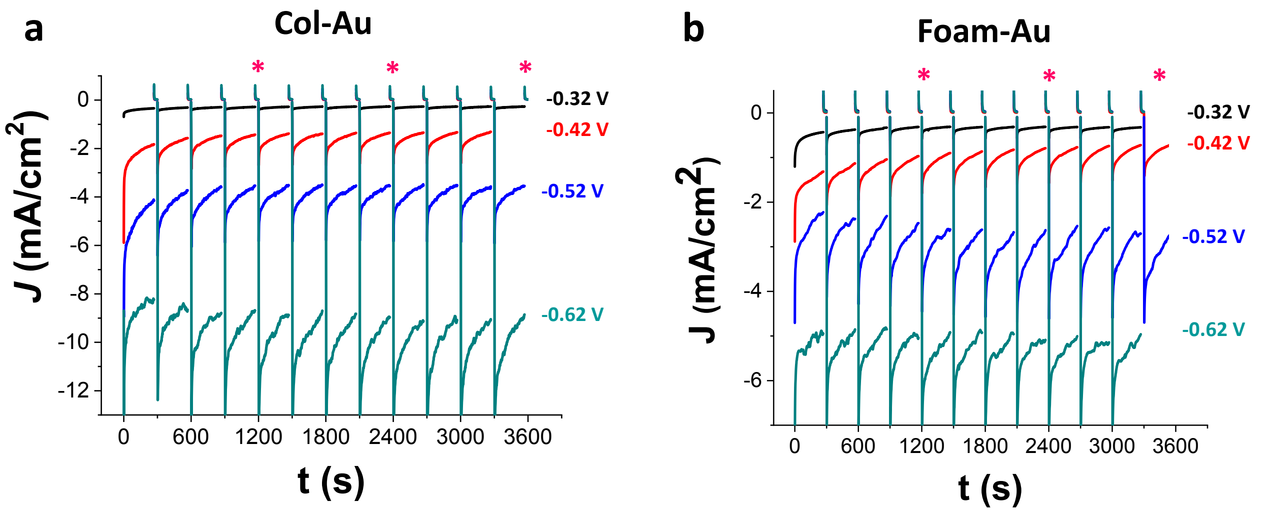

Figure S5. Stepped chronoamperometry experiments performed for the accumulation of the products obtained with Col-Au **(a)** and Foam-Au **(b)** respectively. In particular, the cathodes were stepped between the bias reported in the graphs (for 270 s) and the OCP (for 30 s). The sampling timings are indicated with pink stars and correspond to 1200, 2400 and 3600 s delays from the beginning of each experiment.

Table S2. Faradic efficiency for the two main products (H_2 and CO) obtained with Foam-Au and Col-Au cathodes, as well as with an Au foil, at different applied biases (also expressed in terms of overpotential for the two different reactions). The H_2/CO ratio is also reported.

Cathode	Applied bias, V vs RHE	FE_{H2}, % (η_{H2}, V)	FE_{CO}, % (η_{CO}, V)	H_2/CO ratio
Foam-Au	-0.42	81 ± 3 (0.42)	13 ± 1 (0.31)	6.2
	-0.52	86 ± 2 (0.52)	7 ± 1 (0.41)	12.3
	-0.62	80 ± 3 (0.62)	6 ± 3 (0.51)	14.5
Col-Au	-0.42	62 ± 18 (0.42)	35 ± 7 (0.31)	1.8
	-0.52	69 ± 3 (0.52)	31 ± 3 (0.41)	2.2
	-0.62	83 ± 6 (0.62)	10 ± 3 (0.51)	8.3
Au foil	-0.52	85 ± 5 (0.52)	4 ± 1 (0.41)	23.8
	-0.62	65 ± 6 (0.62)	3 ± 1 (0.51)	19.1
	-0.72	68 ± 5 (0.72)	6 ± 1 (0.61)	10.8

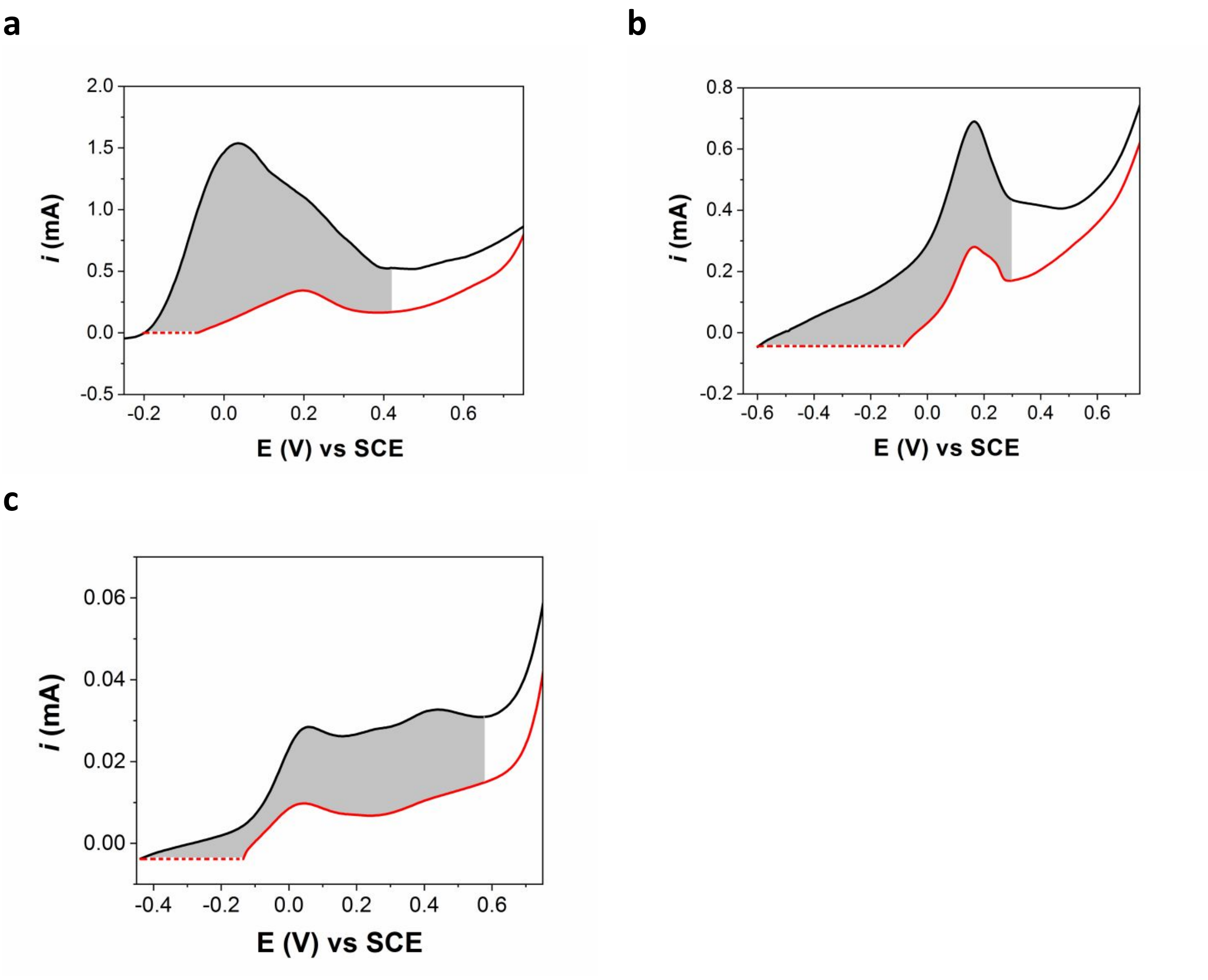

Figure S6. CO_{bridge} stripping analysis for the Col-Au **(a)**, Foam-Au **(b)** and Au foil **(c)** cathodes. The first linear scans (black traces) were recorded immediately after the corresponding bulk electrolyses (pulsed between –0.42 V vs RHE and OCP). For all the cathodes, the charge due to the CO_{bridge} stripping was calculated by integrating the 1st scan over the potential range indicated with the gray area (*i.e.* before Au oxidation starts to occur), using the corresponding 3rd scan (red trace) as the baseline, and dividing the obtained value by the scan rate (0.05 V/s). The resulting CO_{bridge} stripping charges are reported in Table S3, together with the corresponding CO_{bridge} surface concentrations.

Table S3. CO_{bridge} stripping charges calculated from Figures S6a-c for the Col-Au, Foam-Au and Au foil cathodes. The corresponding concentrations of CO_{bridge} spectators stripped from the electrodic surfaces are also reported, considering that the stripping process is bielectronic.

**Cathode**	**CO_{bridge} stripping charge (mC)**	**CO_{bridge} surface concentration, for geometric area (nmol/cm^2)**	**CO_{bridge} surface concentration, for ECSA area (nmol/cm^2)**
Col-Au	9.8	42.3	4.0
Foam-Au	3.7	25.6	2.8
Au foil	0.27	1.4	1.4

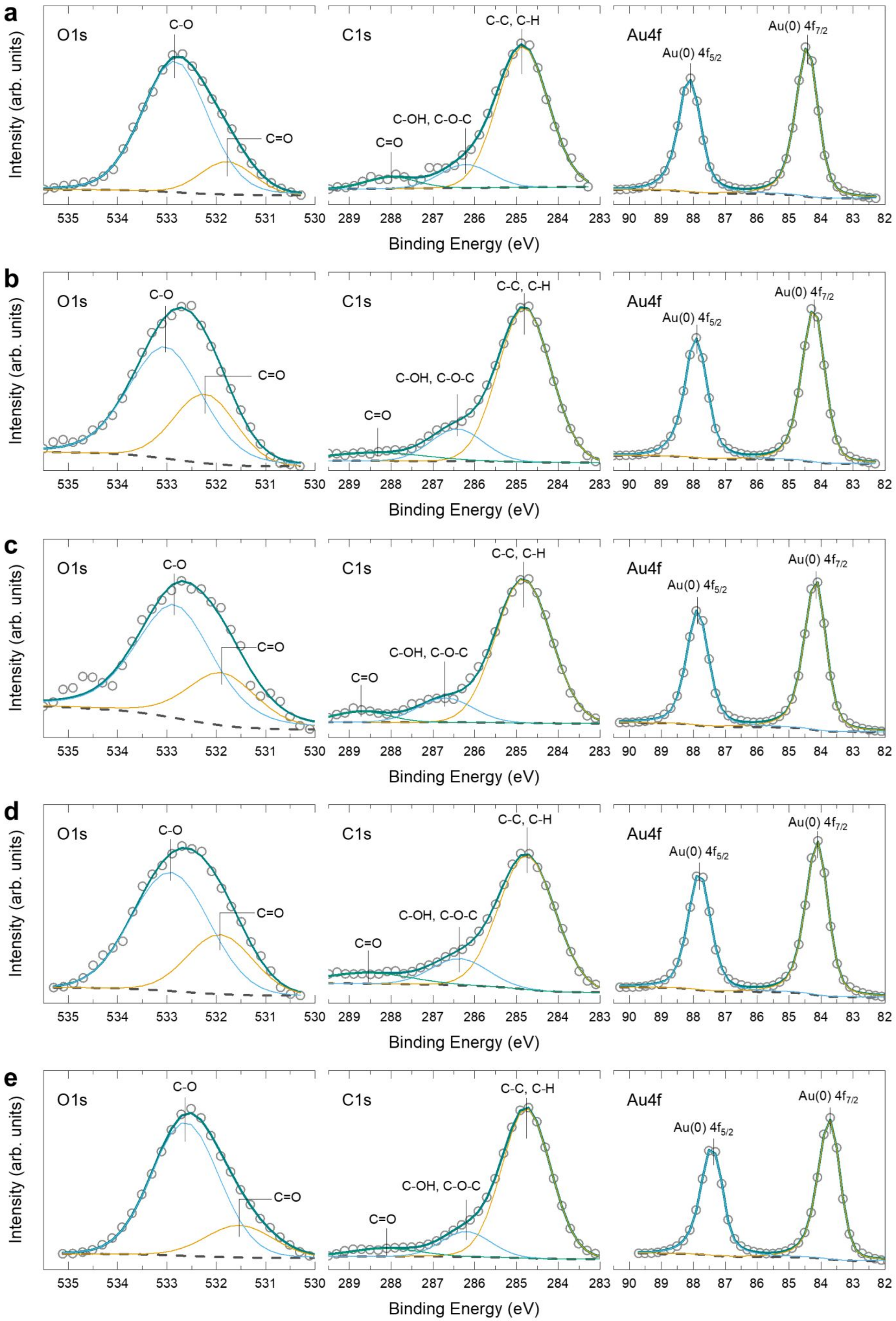

Figure S7. High-resolution O1s (left), C1s (center) and Au4f (right) XPS spectra of **(a)** pristine Col-Au, **(b)** tested Col-Au, **(c)** pristine Foam-Au, **(d)** tested Foam-Au, and **(e)** tested Au foil (circles: experimental spectra; thick solid lines: cumulative fit; thin solid lines: individual peaks; dashed lines: background).

Table S4. Details on XPS peak fitting.

Sample	% at. Au	% at. C	% at. O	Region	Band	Pos. (eV)
Pristine Col-Au	46.5	40.1	13.4	O1s	C-O	532.84
					C=O	531.78
				C1s	C=O	288.00
					C-OH, C-O-C	286.22
					C-C, C-H	284.87
				Au4f	Au(0) $4f_{5/2}$	88.13
					Au(0) $4f_{7/2}$	84.46
Tested Col-Au	52.8	36.7	10.5	O1s	C-O	533.03
					C=O	532.23
				C1s	C=O	288.32
					C-OH, C-O-C	286.42
					C-C, C-H	284.81
				Au4f	Au(0) $4f_{5/2}$	87.88
					Au(0) $4f_{7/2}$	84.21
Pristine Foam-Au	50.2	41.1	8.8	O1s	C-O	532.85
					C=O	531.89
				C1s	C=O	288.72
					C-OH, C-O-C	286.71
					C-C, C-H	284.83
				Au4f	Au(0) $4f_{5/2}$	87.87
					Au(0) $4f_{7/2}$	84.13
Tested Foam-Au	47.6	40.6	11.8	O1s	C-O	532.93
					C=O	531.93
				C1s	C=O	288.55
					C-OH, C-O-C	286.37
					C-C, C-H	284.75
				Au4f	Au(0) $4f_{5/2}$	87.81
					Au(0) $4f_{7/2}$	84.11
Tested Au foil	19.1	56.2	21.1	O1s	C-O	532.63
					C=O	531.54
				C1s	C=O	288.11
					C-OH, C-O-C	286.21
					C-C, C-H	284.76
				Au4f	Au(0) $4f_{5/2}$	87.36
					Au(0) $4f_{7/2}$	83.71
